# Contagiousness of Leprosy

**Published:** 1914-02

**Authors:** 


Contagiousness of Leprosy. *Public Health Bulletin No. 1,* in an article by George W. McCoy, surgeon, and William J. Goodhue, M. D., "The Danger of Association With Lepers at the Molokai Settlement," gives the following conclusions: "Of 119 men, practically all Hawaiians or persons of mixed Hawaiian blood, living in the same house with lepers, five (4.2 per cent.), developed leprosy. Of 106 women, practically all Hawaiians or persons of mixed Hawaiian blood, living in the same house with lepers, five (4.71 per cent.), developed leprosy. Of 12 women, all Caucasians, who lived in such contact with lepers as is necessary in administering to their bodily and spiritual needs, none developed the disease. Of 23 men, all Caucasians, who lived in such contact with lepers as is necessary in administering to their bodily and spiritual needs, three (13 per cent.), developed the disease. So far as we could ascertain, the shortest period in which the disease developed after the person entered the settlement was three years (2 cases), and the longest 17 years.

